# Data describing inhibitory profiles of sugars against hemagglutination by the botulinum toxin complex of *Clostridium botulinum* serotypes C and D

**DOI:** 10.1016/j.dib.2016.09.014

**Published:** 2016-09-17

**Authors:** Yoshimasa Sagane, Minoru Ito, Keita Miyata, Tomonori Suzuki, Koichi Niwa, Suguru Oguri, Toshihiro Watanabe

**Affiliations:** a Department of Food and Cosmetic Science, Faculty of Bioindustry, Tokyo University of Agriculture, 196 Yasaka, Abashiri 099-2493, Japan; b Groupwide Research and Development, Noevir Co., Ltd., 112-1, Okadacho, Higashiohmi 527-8588, Japan; c Department of Nutritional Science and Food Safety, Faculty of Applied Bioscience, Tokyo University of Agriculture, 1-1-1 Sakuragaoka, Setagaya-ku, Tokyo 156-8502, Japan; d Department of Bioproduction, Faculty of Bioindustry, Tokyo University of Agriculture, 196 Yasaka, Abashiri 099-2493, Japan

**Keywords:** *Clostridium botulinum*, Hemagglutinin, Sugar recognition, Botulinum toxin complex

## Abstract

Serotype C and D of *Clostridium botulinum* produce botulinum toxin complex (TC), which is comprised of botulinum neurotoxin, nontoxic nonhemagglutinin, and hemagglutinins (HAs). The TC is capable of aggregating equine erythrocytes via interaction between one of the HAs, namely HA-33, and sugar chains on the cell surface. This hemagglutination is inhibited by specific sugars. In this data article, we used four TCs from serotype C and D strains. The hemagglutination-inhibiting effects of 18 sugars and 8 glycoproteins were studied. The purified TC was mixed with the sugar to enable binding of the sugar to the TC; then, the erythrocytes were added to the mixture. Specific binding between the sugar and TC resulted in inhibition of cell aggregation. Here, data illustrating the inhibitory effects of various sugars and glycoproteins against hemagglutination induced by TC of *C. botulinum* serotypes C and D are presented.

**Specifications Table**

**Table:** 

Subject area	Biology
More specific subject area	Microbiology
Type of data	Table
How data was acquired	Protein purification, SDS-PAGE, hemagglutination assay, and hemagglutination inhibition assay
Data format	Raw
Experimental factors	Toxin complexes produced by four strains of *C. botulinum* were purified from the culture media of each strain.
Experimental features	Inhibitory effects of sugars and glycoproteins against hemagglutination induced by the toxin complex were assessed.
Data source location	Abashiri, Japan
Data accessibility	Data are presented in this article

**Value of the data**

1. The data presented illustrate the specific inhibitory effect exerted by 18 sugars and 8 glycoproteins on hemagglutination by the toxin complex (TC) of serotypes C and D of *C. botulinum*.
2. The results in this article indicate that two modes of hemagglutination mediated by the TCs of *C. botulinum* serotypes C and D strains may be distinguished via the inhibitory profiles exerted by the present sugars and glycoproteins.
3. The data in this article should be useful for elucidation of the mechanism underlying the recognition of sugars by the TCs of *C. botulinum* serotypes C and D.

## 1 Data

Here, we present data illustrating the inhibitory effects of 18 sugars and 8 glycoproteins (listed in Table 1) on hemagglutination by L-TC, a complex of the botulinum neurotoxin (BoNT), nontoxic nonhemagglutinin (NTNHA), and three types of hemagglutinin subcomponents (HA-70, HA-17, and HA-33), produced by the *C. botulinum* serotype C strain Stockholm (C-St), Yoichi (C-Yoichi), D strain CB-16 (D-CB16), and 1873 (D-1873).

## 2 Experimental design

L-TC was purified from the culture of *C. botulinum* C-St [1], C-Yoichi [2], D-CB16 [3], and D-1873 [4] as described in the cited studies. Of the L-TC components, the BoNT also binds to the cells via sugar-recognition ability [5]. However, the residues essential for the sugar-recognition in the BoNT molecule are covered with other components of the L-TC [6], [7]; thus the cell binding of the L-TC dominantly depend on the HA-33 components that exposed to the most outside of the molecule [7]. To date, it has been shown that, in the serotypes C and D of *C. botulinum,* there are at least two types of L-TC containing the HA-33 protein, that preferentially recognize sugar chains with sialic acid moieties (of C-St and D-CB16) or galactose moieties (of C-Yoichi and D-1873) at their termini [2], [4]. Previous studies have shown that C-St and D-CB16 L-TCs exert the hemagglutination activity against equine erythrocytes, whereas C-Yoichi and D-1873 L-TCs exhibited no, or very low, hemagglutination titer against equine erythrocytes. However, C-Yoichi and D-1873 L-TCs were found to exhibit hemagglutination titer against equine erythrocytes treated with neuraminidase [2], [4]. Therefore, normal erythrocytes were used to study hemagglutination by C-St and D-CB16, and neuraminidase-treated erythrocytes were used to study hemagglutination by the C-Yoichi and D-1873 strains.

## 3 Materials and methods

### 3.1 SDS-PAGE

SDS-PAGE was performed as described by Laemmli [8], by using a 13.6% polyacrylamide gel with 2-mercaptoethanol.

### 3.2 Preparation of equine erythrocyte suspension

Erythrocytes in defibrinated equine blood (Kojin Bio, Sakaido, Saitama, Japan) were collected by centrifugation ( 1000×*g*, 5 min, 4 °C) (TOMY GRX-220, Tomy Seiko Co., Ltd., Tokyo, Japan) and rinsed with 0.15 M phosphate buffer, pH 7.0. Suspension of the erythrocytes (1%) was prepared with the same buffer. Erythrocytes were treated with *Clostridium perfringens* neuraminidase (Sigma-Aldrich). Neuraminidase (0.5 U) was added to a 10% equine erythrocyte suspension, and the mix was incubated at 37 °C for 1 h. Treated erythrocytes were collected, rinsed, and suspended in 0.15 M phosphate buffer (pH 7.0) to create a 1% suspension.

### 3.3 Hemagglutination assay and inhibitory test against hemagglutination

Hemagglutination assay was performed by the microtitration method, using a multiwell titer plate (Nunc A/S, Roskilde, Denmark). Samples (35 µl) of each preparation (100 µg/ml) were diluted in serial two-fold steps with 0.15 M phosphate buffer (pH 7.0) and mixed with an equal volume of a 1% equine erythrocyte suspension. After incubation at 20–25 °C for 2 h, the reciprocal hemagglutination titer was determined as 2*n*.

For the inhibitory test, sugars (d-glucose, d-mannose, galactose, *N*-acetyl-d-glucosamine, methyl-α-d-mannoside, l-fucose, xylose, fructose, rhamnose, *N*-acetyl-d-galactosamine, sucrose, *N*-acetylneuraminic acid, maltose, cellobiose, lactose, chitin oligomers, *N*-acetyllactosamine, and chitobiose) and glycoproteins (bovine fetuin, asialofetuin, ovomucoid, ovoalbumin, human transferrin, horseradish peroxidase, yeast invertase, and human α1-acid glycoprotein) were used in this study. The L-TC solution (10 µl, titer 2) was incubated with an equal volume of sugar or glycoprotein solution (final concentration of 5 µg/ml), at room temperature, for 1 h. Thereafter, the solution was incubated with 20 µl of equine erythrocyte suspension (1%) at 37 °C for 1 h.

## Figures and Tables

**Table 1:** Inhibitory effects on hemagglutination by C-St, C-Yoichi, D-CB16, and D-1873 L-TC.

Sugars or glycoproteins	C-St	D-CB16	C-Yoichi^a^	D-1873^a^
d-glucose	–	–	+	+
d-mannose	–	–	+	+
Galactose	–	–	+	+
*N*-acetyl-d-glucosamine	–	–	–	–
Methyl-α-d-mannoside	–	–	+	+
l-Fucose	–	–	+	+
Xylose	–	–	–	–
Fructose	–	–	+	+
Rhamnose	–	–	+	+
*N*-Acetyl-d-galactosamine	–	–	+	+
Sucrose	–	–	+	+
*N*-Acetylneuraminic acid	+	+	–	–
Maltose	–	–	+	+
Cellobiose	–	–	+	+
Lactose	–	–	+	+
Chitin oligomer	–	–	–	–
N-Acetyllactosamine	–	–	+	+
Chitobiose	–	–	–	–
Bovine fetuin	–	–	–	–
Asialofetuin	–	–	+	+
Ovomucoid	–	–	–	–
Ovoalbumin	–	–	–	–
Human transferrin	–	–	–	–
Horseradish peroxidase	–	–	–	–
Yeast invertase	–	–	+	+
Human α1-Acid glycoprotein	+	+	–	–

a Neuraminidase-treated erythrocyte was used.
